# Social Defeat Stress Decreases mRNA for Monoamine Oxidase A and Increases 5-HT Turnover in the Brain of Male Nile Tilapia (*Oreochromis niloticus*)

**DOI:** 10.3389/fphar.2018.01549

**Published:** 2019-01-09

**Authors:** Yuki Higuchi, Tomoko Soga, Ishwar S. Parhar

**Affiliations:** Brain Research Institute, Jeffrey Cheah School of Medicine and Health Sciences, Monash University Malaysia, Bandar Sunway, Malaysia

**Keywords:** stress, serotonin, 5-HIAA, MAO, depression, tilapia, neurotransmitter

## Abstract

Stress induces various neurobiological responses and causes psychiatric disorders, including depression. Monoamine oxidase A (MAO-A) plays an important role in various functions of the brain, such as regulation of mood, anxiety and aggression, and dysregulation of MAO-A is observed in stress-related psychiatric disorders. This study addressed the question whether acute social stress induces changes to transcriptional and/or post-transcriptional regulation of MAO-A expression in the brain. Using male Nile tilapia (*Oreochromis niloticus*), we investigated whether acute social stress, induced by the presence of a dominant male fish, changes the expression of MAO-A. We measured gene expression of MAO-A by quantitative PCR, enzymatic activity of MAO-A by the luminescent method, and 5-HT and 5-HIAA levels by liquid chromatography–mass spectrometry in the brain of socially stressed and control fish. Socially stressed males showed decreased MAO-A mRNA levels, consistent MAO-A enzymatic activity, increased 5-HT turnover in the brain, and elevated plasma cortisol levels, compared to controls. Our results suggest that acute social stress suppresses the transcription of MAO-A gene, enhances 5-HT metabolism but does not affect the production of MAO-A protein.

## Introduction

Stress elicits various neurobiological responses and causes several psychiatric disorders, including depression, anxiety disorder, and post-traumatic stress disorder (Lucassen et al., 2014; Chiriţã et al., 2015). Dysregulation of monoamine oxidase A (MAO-A) is observed in stress-related psychiatric disorders. Elevated MAO-A activity and 5-hydroxytryptamine (5-HT) degradation in the brain are well-known features in depressed patients and animals exposed to chronic stress. Positron emission tomography shows higher density of MAO-A in the brain of patients with major depressive disorders, compared to healthy subjects (Meyer et al., 2006). Higher MAO-A density in the brain is associated with the recurrence of depressive symptoms (Meyer et al., 2009). In addition, chronically stressed animals show increased MAO-A levels, decreased 5-HT levels, and elevated 5-hydroxyindoleacetic acid (5-HIAA) levels (Winberg and Lepage, 1998; Grunewald et al., 2012).

Monoamine oxidase A is an enzyme that catalyzes the degradation of 5-HT to 5-HIAA. Increased MAO-A activity, therefore, reduces 5-HT levels in the brain. Degradation of 5-HT to 5-HIAA by MAO-A leads to the production of hydrogen peroxide, one of reactive oxygen species, which causes a decline of cellular functions (Maurel et al., 2003; Fitzgerald et al., 2014). In addition, the regulation of MAO-A is associated with the hypothalamus-pituitary-adrenal (HPA) axis. A selective glucocorticoid receptor agonist, dexamethasone, increases MAO-A expression by activating the transcription of MAO-A gene (Ou et al., 2006). Understanding the regulation of MAO-A during stress and finding an endogenous mechanism to reduce MAO-A levels would be important to develop a new approach to control MAO-A for treatment of stress-related psychiatric disorders.

Various species and stressors are used to test the influence of stress on animals. Chronic stress model contributes to the understanding of neurobiological differences between normal and depression-like profile; however, due to long exposure period, the endogenous mechanism to prevent further exaggeration of negative stress response might be missed (Musazzi et al., 2017). Therefore, this study uses acute stress to observe immediate responses of MAO-A regulation. Acute stress may induce physiological responses for the maintenance of normal biological functions of the brain (McEwen, 2004, 2008; Edwards et al., 2007; Sinyakov et al., 2017). Biological response to stress may vary depending on the duration of stress exposure (Øverli et al., 1999; Chen and Fernald, 2011; Nardocci et al., 2014). For example, stress responses in the 5-HT system and the HPA axis vary depending on the duration and frequency of stressors (Keeney et al., 2006; Moltesen et al., 2016).

In this study, using male Nile tilapia (*Oreochromis niloticus*), we studied whether acute social stress induces responses in the regulation of MAO-A by measuring the gene expression and enzymatic activity of MAO-A, and 5-HT turnover. Nile tilapia shows changes in body color (Corrêa et al., 2003), eye color (Volpato et al., 2003; Vera Cruz and Brown, 2007), and behavior (Corrêa et al., 2003) in response to stressors. Darkening of body color is an indicator of stress in Nile tilapia. Socially stressed fish shows darker body color, compared to the fish in higher hierarchical rank (Fernandes and Volpato, 1993; Corrêa et al., 2003). Nile tilapia are social animals, which show clear social hierarchy in the male conspecific group, as some other mammalian species do. It also has cortisol as a major endogenous glucocorticoid, as do humans. Therefore, we adopted this species as a model animal of acute social stress.

## Materials and Methods

### Experimental Animals

Male Nile tilapia used in this study were obtained from Inter Sea Fishery (Malaysia) Sdn Bhd or grown at the fish facility, Monash University Malaysia. Adult fish (4–8 months old) were group-housed in a home tank (90 cm × 45 cm × 45 cm) under 14 h light/10 h dark cycles. Male fish showing typical body color (silver) and behavioral pattern (aggression) of male Nile tilapia of high social status were used as fish going to play a dominant role in the social defeat stress procedure described below. The fish going to play a dominant role were kept in individual tanks (36 cm × 22 cm × 26 cm) before social defeat procedure. Fish were fed with commercial dry pellets (TP-1, Star Feedmills, Malaysia) three times per day. All the procedures for animal care and experiments in this study were approved by Monash University Animal Ethics Committee (MARP/2015/180).

### Social Defeat Stress

In this study, socially stressed animals were defined as male fish which showed body color darkening, while control fish as male fish which showed neither aggressive/submissive behavior nor body color changes. In the social defeat stress experiment, the fish going to play a dominant role (15–16 cm standard length) was placed in an experimental tank (36 cm × 22 cm × 26 cm) for 1 h (9:00–10:00 h) for acclimatization. Test fish (11–12 cm standard length) was captured from the home tank, kept in the experimental tank from 10:00 to 12:30 or 15:00 h. Immediately after the exposure to social defeat stress, defeated fish were anesthetized in 0.02% benzocaine (Sigma-Aldrich, MO, United States) for approximately 1–2 min until the fish were immobilized. Blood samples were collected from a caudal vein of the fish, using 1 mL heparinized syringes and 25G syringe needles. The brain was removed and immediately dissected into three parts (Area I, telencephalon and preoptic area; Area II, optic tectum, midbrain, and hypothalamus; Area III, cerebellum and hindbrain; Figure 2A), frozen on dry ice, and stored at -80°C until analysis. Video recording during stress exposure was used to check the behavior of fish. Control fish were captured from the group-housed tank. Samples from control fish corresponding to 2.5 and 5.0 h stress groups were collected at 12:30 and 15:00 h, respectively, using the same sampling methods as stress groups.

### Measurement of Plasma Cortisol Levels

The experimental condition for the high performance liquid chromatography (HPLC) was adopted from a previous study (Thang et al., 2016) with minor modification. Plasma samples were separated from blood samples by centrifugation at 3000×*g* for 10 min. Cortisol was extracted from the plasma using the following procedure: dexamethasone (50 ng; internal standard; Nacalai Tesque, Kyoto, Japan) and dichloromethane (400 μL; Fisher Scientific UK Ltd., Loughborough, United Kingdom) were added to plasma (200 μL) in microcentrifuge tubes. The tubes were tightly capped, vortex mixed for 15 min and centrifuged at 15000 rpm for 10 min. The organic layer was transferred to a new centrifuge tube. Dichloromethane (400 μL) was added to the remaining aqueous phase and the extraction was repeated. After the organic phase was collected in the same centrifuge tube, the sample was washed with 0.1 M NaOH (100 μL). The organic layer was separated by centrifugation at 2000 ×*g* for 5 min and transferred into a new tube; then the solvent was evaporated at 50°C. The residue was reconstituted with 55% methanol in water (100 μL), filtered with Cosmospin filter G (hydrophilic PTFE membrane filter with 0.2 μm pores, Nacalai Tesque) and used for analysis. A stock solution of cortisol was prepared by dissolving 10 mg of cortisol powder (Nacalai Tesque) in 10 mL of methanol (Fisher Scientific UK Ltd.). Calibration solutions (5, 10, 50, 100, 200, 300, 400, and 500 ng/mL of cortisol) were prepared in 55% methanol with dexamethasone (500 ng/mL; internal standard). 20 μL of each sample was injected manually into HPLC-DAD system (1260 Infinity, Agilent Technologies, CA, United States). The separation was performed with Agilent Zorbax Eclipse Plus C18 Rapid Resolution (4.6 mm × 75 mm, 1.8 μm, Agilent Technologies) maintained at 30°C and mobile phase consisted of methanol-water (55/45; v/v). Flow rate of the mobile phase was 0.8 mL/min. Cortisol and dexamethasone were detected and quantified by UV absorbance at 245 nm. Peak areas of cortisol and dexamethasone on the chromatograms were calculated with ChemStation software (RRID:SCR_015742, Agilent Technologies).

### Gene Expression Analysis

Total RNA was extracted from the brain samples with Favorprep Tri-RNA reagent (Favorgen Biotech Corporation, Taiwan), following the manufacturer’s protocol. The extracted total RNA (1000 ng for each brain sample) was transcribed into cDNA using High-Capacity cDNA Reverse Transcription Kit (Applied Biosystems, CA, United States). The cDNA samples were stored at -20°C until analysis. Quantitative real-time PCR (qPCR) was performed to measure the expression levels of MAO-A and β-actin (reference gene) mRNA using 7500 Fast Real-time PCR system (Applied Biosystems, CA, United States) with SensiFAST^TM^ SYBR Hi-ROX Kit (Bioline Reagents, United Kingdom). The plasmid containing MAO-A or β-actin fragment was serially diluted to a concentration of 10^2^, 10^3^, 10^4^, 10^5^, 10^6^, 10^7^, and 10^8^ copy/μL as a standard DNA for absolute quantification of mRNA. Reaction conditions were 95°C for 2 min, 40 cycles at 95°C for 5 s, and 60°C for 30 s followed by dissociation steps. Specific Primers for MAO-A and β-actin were used and designed as follows: MAO-A, 5′-AGCTGCTGGAGGGCTACTAA-3′ (forward) and 5′-TTCCATCCAACACGGTCTGC-3′ (reverse); β-actin, 5′-CACCGTGCTGTCTGGAGGTA-3′ (forward) and 5′-TTACGCTCAGGTGGGGCAA-3′ (reverse). The data was acquired and analyzed with 7500 Real-Time PCR Software (RRID:SCR_014596, Applied Biosystems), using ΔΔCt method.

### Measurement of MAO-A Enzymatic Activity

Enzymatic activity of MAO-A in the brain was measured with an MAO-Glo Assay kit (Promega, WI, United States), following the manufacturer’s protocol. Brain tissue samples were homogenized in 10-fold volume of 100 mM HEPES-KOH buffer (pH 7.6) containing glycerol (5%) and protease inhibitor cocktail (Nacalai Tesque) and centrifuged at 600×*g* for 10 min. 3 μL of the supernatant was used for each MAO-A reaction. The luminescent signal was measured by a microplate reader (Infinite M200 PRO; Tecan, Switzerland).

### Measurement of 5-HT and 5-HIAA

Measurement of 5-HT and 5-HIAA was performed with liquid chromatography–mass spectrometry (LC-MS). Brain tissues were homogenized in 350 μL of 50% acetonitrile (Fisher Scientific UK Ltd.) with 0.1% formic acid (Sigma-Aldrich) and 20 ng/mL of isoproterenol (internal standard; Nacalai Tesque) in microcentrifuge tubes, incubated for 5 min on ice and centrifuged at 15000 rpm for 15 min for protein precipitation. The supernatant was transferred into Cosmospin filter G (hydrophilic PTFE membrane filter with 0.2 μm pores, Nacalai Tesque), centrifuged at 5000 ×*g* for 10 min and used for analysis. Calibration solutions containing 1.25, 2.5, 5, 10, 20, 40, 80, 160 ng/mL of 5-HT (Sigma-Aldrich) and 5-HIAA (Sigma-Aldrich), and 20 ng/mL of isoproterenol (internal standard) were prepared in 50% acetonitrile with 0.1% formic acid. The measurement was performed with an Agilent Technologies 6410 Triple Quad LC/MS equipped with a Zorbax SB-C18 column (Narrow-Bore, 2.1 × 150 mm, 3.5 μm column; Agilent Technologies). The mobile phase was a mixture of water with 0.1% formic acid (A) and acetonitrile with 0.1% formic acid (B). The elution was as follows: 5% B from 0 to 0.3 min; 5% B to 50% B from 0.3 to 2.5 min; 50 to 100% B from 2.5 to 3.0 min. The flow rate was 0.5 mL/min. The mass spectrometer was operated in the positive-ion mode. The peaks of 5-HT, 5-HIAA, and isoproterenol were monitored by the product ion (m/z 177.1 → 160 for 5-HT, m/z 192.07 → 146 for 5-HIAA, and m/z 212.1 →194 for isoproterenol). Data acquisition and calculation were performed with Agilent Masshunter Quantitative Analysis software (RRID:SCR_015040, Agilent Technologies).

### Statistics

Statistical differences were evaluated using Welch’s *t*-test for comparison of two groups. One-way ANOVA was followed by Tukey’s honest significant difference test for multiple comparisons. A value of *p* < 0.05 was considered statistically significant.

## Results

### Social Stress Changed Body Color of Fish and Increased Plasma Cortisol Levels

**FIGURE 1:**
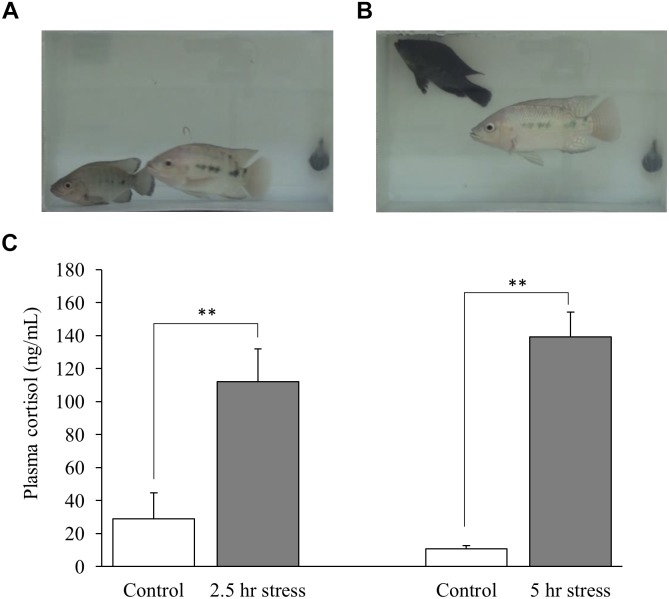
Changes in body color of fish **(A,B)** and plasma cortisol levels **(C)**. **(A)** Focal fish (bottom left) showed normal body color at the beginning of stress exposure. **(B)** Focal fish (top left) showed darker body color after stress exposure. **(C)** Plasma cortisol levels were significantly elevated by social stress. Fish in 2.5 and 5 h stress groups were exposed to stress from 10:00–12:30 to10:00–15:00 h, respectively. Blood sampling from fish in the control group corresponding to each stress group was conducted at 12:30 or 15:00 h, respectively. *n* = 6 for control (12:30 h); *n* = 8 for 2.5 h stress; *n* = 9 for control (15:00 h); *n* = 4 for 5 h stress. ^∗∗^*p* < 0.01.

In social defeat stress experiment, the body color darkening was observed in all the socially stressed fish. An example of social stress-induced body color changes is shown in Figures 1A,B. Plasma cortisol levels were significantly higher in social stress group, compared to control groups [*p* < 0.01 for 2.5 h social stress (111.9 ng/mL) vs. control (28.9 ng/mL); *p* < 0.01 for 5 h social stress (139.3 ng/mL) vs. control (10.7 ng/mL); Figure 1C].

### Social Stress Downregulates MAO-A Gene Expression

**FIGURE 2:**
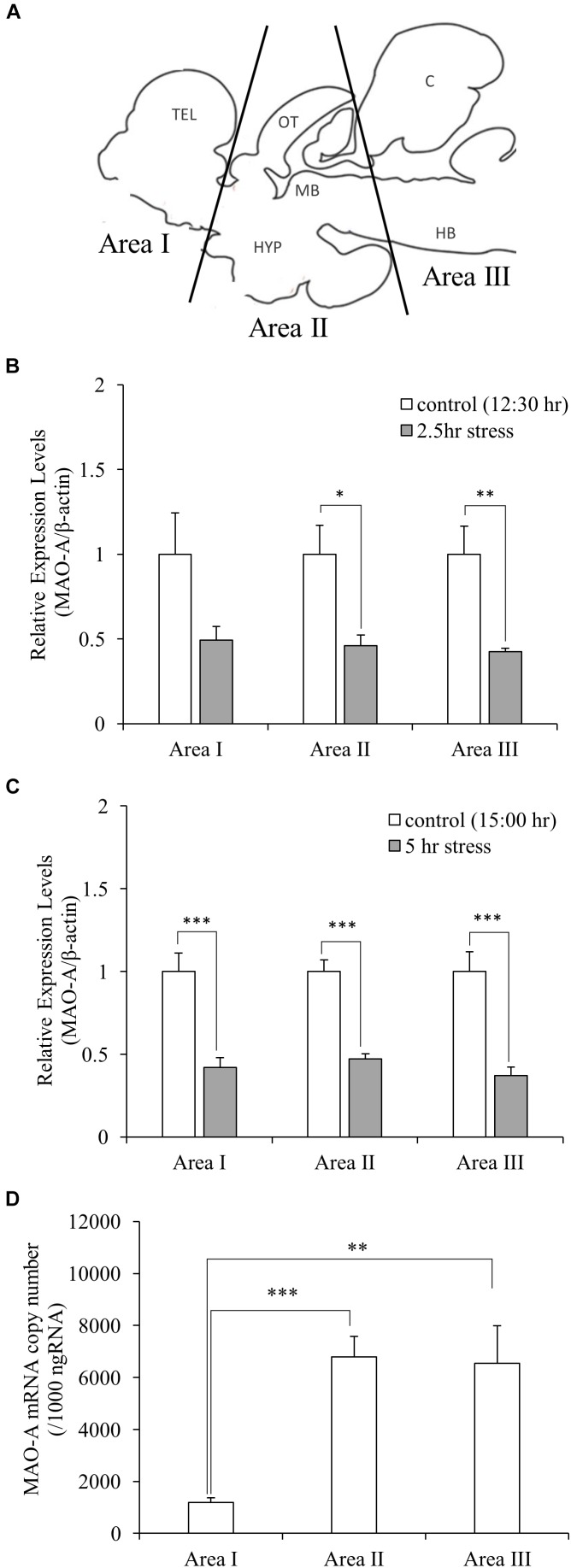
**(A)** Dissection of the brain samples. TEL, telencephalon; OT, optic tectum; MB, midbrain; HYP, hypothalamus; C, cerebellum; HB, hindbrain. (**(B,C)** Relative gene expression levels of MAO-A. MAO-A expression levels in the brain were decreased by social stress, except for brain area I after 2.5 h stress. **(D)** Copy number of MAO-A mRNA in the brain. MAO-A gene was expressed mainly in Area II and III. *n* = 10/group ^∗^*p* < 0.05, ^∗∗^*p* < 0.01, ^∗∗∗^*p* < 0.001.)

Gene expression of MAO-A was measured by qPCR. Gene expression levels of MAO-A in the brain were significantly decreased by social stress both in 2.5 and 5.0 h stress groups, compared to control groups, except for brain area I after 2.5 h social stress [0.49, 0.46, 0.42-fold (2.5 h stress vs. control); 0.42, 0.47, 0.37-fold (5.0 h stress vs. control) in the brain area I, II, and III, respectively, (Figures 2B,C)]. In addition, significant differences in MAO-A mRNA levels were detected between brain areas [one-way ANOVA, *F*(2,27) = 10.88, *p* < 0.001]. MAO-A mRNA is mainly localized in brain area II and III [1.19 × 10^3^± 1.8 × 10^2^, 6.78 × 10^3^± 8.0 × 10^2^, and 6.54 × 10^3^± 1.4 × 10^3^ copies/μg total RNA in Area I, II, and III, respectively. *p* < 0.001 for Area I vs. II; *p* < 0.01 for Area I vs. III; *p* = 0.98 for Area II vs. III (Figure 2D)].

### MAO-A Enzymatic Activity

**FIGURE 3:**
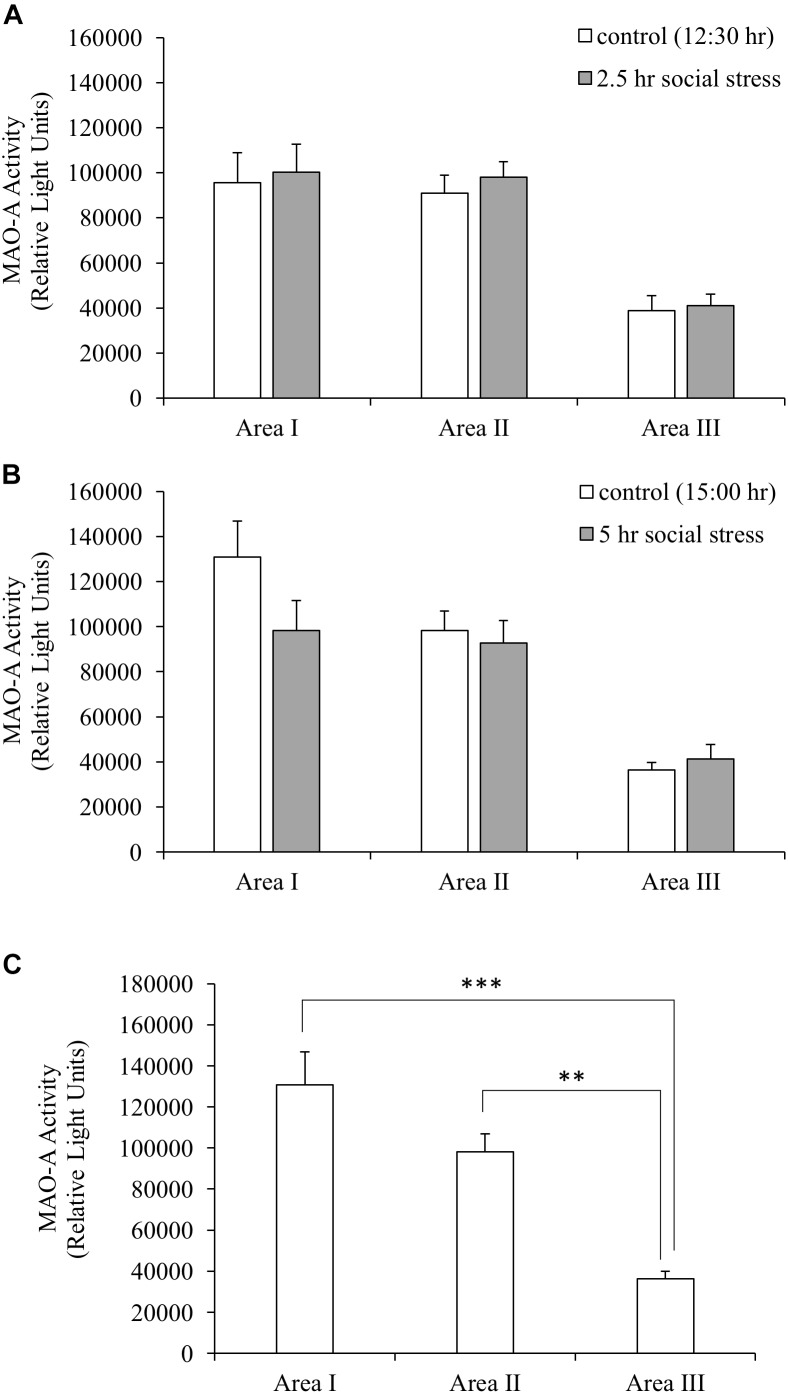
**(A,B)** MAO-A enzymatic activity. MAO-A enzymatic activity in the brain was not changed by social stress. **(C)** MAO-A enzymatic activity in the brain (control group sampled at 15:00 h). MAO-A enzymatic activity in Area I and II were significantly higher than that of Area III. *n* = 10/group ^∗∗^*p* < 0.01, ^∗∗∗^*p* < 0.001.

Monoamine oxidase A enzymatic activity was measured to understand whether stress-induced decreased MAO-A gene expression caused changes in MAO-A enzymatic activity. MAO-A enzymatic activity did not change following social stress in the brain areas tested (Figures 3A,B). Significant differences in MAO-A enzymatic activity were found between different brain regions (one-way ANOVA, *F*(2,27) = 19.98, *p* < 0.001). Area I and II showed significantly higher MAO-A enzymatic activity, compared to Area III (*p* < 0.001 for Area I vs. III; *p* < 0.01 for Area II vs. III; Figure 3C).

### Social Stress Increased 5-HT Turnover in the Brain

The influences of social stress on 5-HT, 5-HIAA, and 5-HT turnover were tested in three brain areas (Figure 2A). 5-HIAA levels were significantly increased (1.56, 1.63, and 2.17-fold in the brain area I, II, and III, respectively) by 5 h social stress exposure, whereas 5-HT levels remained unchanged (Table 1). In parallel with the increases in 5-HIAA levels, 5-HIAA/5-HT ratios were significantly increased [1.88, 1.82, and 2.27-fold in area I, II, and III, respectively, (Table 1)].

**Table 1:** Changes in 5-HT, 5-HIAA, and 5-HIAA/5-HT ratios induced by social stress.

Parameter	Brain area	Control group	Stress group	Fold change (stress/control)	*p*-value	95% CI	*n*
5-HT	I	232.12 ± 9.56	196.64 ± 16.39	0.85	0.088	-77.11 to 6.15	8/group
	II	221.50 ± 29.19	174.02 ± 17.65	0.79	0.19	-122.16 to 27.20	8/group
	III	87.70 ± 10.90	74.91 ± 6.93	0.85	0.34	-40.96 to 15.38	8/group
5-HIAA	I	105.40 ± 5.70	164.67 ± 23.11	1.56	0.038	4.20 to 114.34	8/group
	II	102.91 ± 3.64	167.79 ± 12.61	1.63	0.0011	34.72 to 95.03	8/group
	III	38.02 ± 2.64	82.55 ± 8.96	2.17	0.0013	23.08 to 65.98	8/group
5-HIAA/5-HT	I	0.46 ± 0.02	0.86 ± 0.12	1.88	0.011	0.12 to 0.68	8/group
	II	0.57 ± 0.12	1.04 ± 0.16	1.82	0.037	0.03 to 0.90	8/group
	III	0.49 ± 0.07	1.11 ± 0.07	2.27	0.000034	0.40 to 0.85	8/group

*Unit for concentrations is ng/g tissue. Numerical data is expressed as mean ± standard error of mean. Statistical significance of the differences between stress group and control group was tested by Welch’s oxt-test. Brain areas I, II, and III are shown in Figure 2A. CI, confidence interval.*

## Discussion

### Social Stress Model of Male Nile Tilapia

In social defeat stress experiment, the test fish was introduced into a tank where fish with a larger body size was placed in advance, and the test fish was forced to experience stress by the presence of the dominant fish. Therefore, this model is similar to learned helplessness model. Socially stressed fish showed clear changes in body color. Previous studies showed stress-induced changes in body color in teleost (Höglund et al., 2000; Barreto and Volpato, 2006; Carpenter et al., 2014), which is consistent with our results in social stress model of male Nile tilapia. In addition, socially stressed fish showed higher cortisol levels in plasma, compared to control fish, indicating that social stress acutely activates the hypothalamus-pituitary-interrenal (HPI) axis, a teleost homolog of mammalian HPA axis. This result is also consistent with previous studies that show high plasma cortisol levels in teleost after exposure to stress (Øverli et al., 2004; Carpenter et al., 2014) and human subject with depression (Carroll et al., 2012).

### Decreased Expression of MAO-A Gene During Social Stress

This study was designed to investigate how acute social stress influences transcriptional and post-transcriptional regulation of MAO-A and 5-HT metabolism in the brain. The expression of MAO-A gene was downregulated in the brain during social stress. This result indicates that MAO-A expression in our social stress model is regulated differently from that of chronic stress models where MAO-A mRNA is increased by chronic social stress (Grunewald et al., 2012; Harris et al., 2015). This result also supports the advantage of the acute stress model in exploring the endogenous mechanism which downregulates the gene expression of MAO-A, which may lead to identification of novel approach to control MAO-A expression. Upregulation of MAO-A gene expression, protein levels and enzymatic activity during depression and chronic stress have been observed in previous studies (Filipenko et al., 2002a,b; Meyer et al., 2006, 2009; Chiuccariello et al., 2014; Sacher et al., 2015). Glucocorticoid is an activator of MAO-A gene expression (Ou et al., 2006). In this study, however, gene expression levels of MAO-A were decreased, while plasma cortisol levels were elevated by social stress, indicating that circulating cortisol is not a determinating factor for MAO-A gene expression during acute stress exposure in our social stress model. The discrepancy between these previous studies and this study might result from the difference in duration of stress. Social stress in this study increased plasma cortisol levels in relatively short period of time (2.5 or 5.0 h), compared to previous studies using chronic stress models (Filipenko et al., 2002a; Grunewald et al., 2012; Harris et al., 2015). The downregulation observed in this study might be a result of stress coping responses of the brain to get the increased 5-HT turnover back to normal levels. In addition to glucocorticoids, expression of MAO-A gene is regulated by transcription factors, such as Krüppel-like factor 11 (Grunewald et al., 2012) and R1 (Chen et al., 2005). Changes in these transcription factors were found in the post-mortem brains of patients with depression (Johnson et al., 2011; Harris et al., 2015). The role of these transcription factors in the acute phase of stress response needs to be investigated to explore the molecular mechanism which decreases MAO-A mRNA during acute stress.

### MAO-A Enzymatic Activity and 5-HT Turnover in the Brain

**FIGURE 4:**
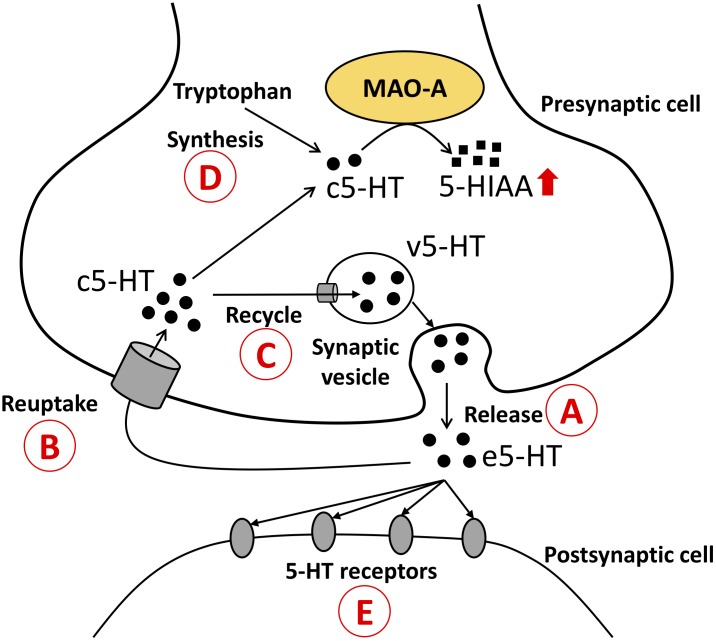
Possible mechanisms that explain the increased 5-HT turnover (5-HIAA/5-HT ratios). 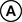 The amount of 5-HT available for MAO-A can be changed by release of 5-HT; 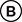 reuptake of 5-HT; 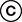 recycle of 5-HT into synaptic vesicles; 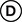 5-HT synthesis in the cells; and 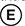 binding of 5-HT with receptors. Possibility 1: Increases in 5-HT release 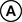 and 5-HT reuptake 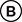 lead to an increase in the supply of c5-HT to MAO-A; thus 5-HT turnover is increased. Possibility 2: Decreased 5-HT recycling into synaptic vesicles 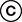 leads to an increase in the supply of c5-HT to MAO-A; thus 5-HT turnover is increased. Possibility 3: Increased 5-HT synthesis 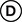 leads to an increase in c5-HT available for MAO-A; thus 5-HT turnover is increased. Possibility 4: Decreased 5-HT receptor 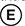 causes an increase in e5-HT, which can be taken up into 5-HT cells, and leads to an increase in the supply of c5-HT to MAO-A; thus 5-HT turnover is increased. 5-HT, 5-hydroxytryptamine; 5-HIAA, 5-hydroxyindoleacetic acid; c5-HT, cytosolic 5-HT; v5-HT, vesicular 5-HT; e5-HT, extracellular 5-HT.

In this study social stress upregulated 5-HT turnover in the brain. This result is consistent with previous studies which tested the influences of chronic social stress on 5-HT turnover (Beitia et al., 2005). However, MAO-A enzymatic activity did not show significant changes, suggesting that increased 5-HIAA levels and 5-HT turnover were not caused by changes in the production of MAO-A protein at the post-transcriptional levels. Enzymatic activity measured in our experiment reflects the total amount of MAO-A protein in the brain tissues. The gap between the increased 5-HT turnover and the unchanged MAO-A enzymatic activity might be explained by changes in the bioavailability of 5-HT (substrate) for MAO-A (enzyme) in the brain. 5-HT needs to be in MAO-A expressing cell to be degraded by MAO-A protein localized on the outer membrane of mitochondria. Several possible explanations can describe the elevated 5-HT turnover in our study. An increase in the release of 5-HT would explain the elevated 5-HIAA and 5-HIAA/5-HT ratios. If 5-HT release and reuptake are increased, the amount of cytosolic 5-HT would be increased; thus 5-HT turnover would also be increased. An increase in 5-HT release in response to stress is strongly supported by previous studies (Keeney et al., 2006; Mo et al., 2008; Mongeau et al., 2010). Reuptake transporters of monoamines, such as serotonin transporter (SERT), organic cation transporters (Gasser, 2006, Gasser et al., 2009; Bacq et al., 2012; Matsui et al., 2016), and plasma membrane monoamine transporter (Engel et al., 2004; Engel and Wang, 2005; Duan and Wang, 2010), may change the availability of 5-HT for MAO-A in the cells and thus affect 5-HT turnover in the brain. Dexamethasone (glucocorticoid receptor agonist) acutely enhances the cell surface localization of SERT in serotonergic neurons derived from mouse embryonic stem cells (Lau et al., 2013), supporting the possibility that SERT might increase the availability of 5-HT for MAO-A and thus increase 5-HT turnover during stress. It is also possible to hypothesize that a decrease in 5-HT recycling into synaptic vesicles could increase the supply of 5-HT to MAO-A, which can lead to an increase in 5-HT turnover. Finally, it is possible to postulate that increases in 5-HT synthesis and/or 5-HT receptors could increases the supply of 5-HT to MAO-A and enhance the 5-HT turnover (Figure 4).

A previous study which tested the impact of confinement stress on MAO in the brain of rainbow trout showed that MAO activity in the telencephalon is initially decreased after 1 h stress exposure but increased again back to normal levels after 3 h stress exposure (Schjolden et al., 2006), indicating that the response of the enzymatic activity can be different depending on the duration of stress. This previous study also reported increases in MAO activity in the optic tectum and hypothalamus, which is different from our results, suggesting that the impact of stress on MAO activity differs depending on the types of stressors or species. The increases in 5-HT turnover observed in this study was similar to those reported in Schjolden et al. (2006), suggesting that the increased 5-HT turnover is a common response to social defeat stress and confinement stress.

### Distribution of MAO-A mRNA and Protein in the Brain

Our data suggest that MAO-A mRNA is mainly distributed in the brain areas including the midbrain, raphe, and hindbrain (Area II and III in Figure 2A) rather than the forebrain (Area I). However, 5-HT turnover was observed in the forebrain as well as other brain areas. In addition, brain area I and II showed higher MAO-A enzymatic activity, compared to area III. These results indicate that MAO-A protein is transported from the posterior regions to the telencephalon. MAO-A protein is localized on the outer membrane of mitochondria. Therefore, the localization of MAO-A is likely to be dependent on mitochondria, which can be transported from soma to terminal through axons (Saxton and Hollenbeck, 2012). Further study will be required to elucidate the mechanism by which the telencephalon recruits MAO-A from other brain regions. The teleost brain has three populations of 5-HT neurons: the raphe, hypothalamus, and pretectal populations (Lillesaar, 2011; Loveland et al., 2014). In zebrafish, MAO is localized mainly in serotonergic and noradrenergic neurons (Anichtchik et al., 2006). The raphe population of 5-HT neurons have projections to the forebrain (Gaspar and Lillesaar, 2012). Therefore, the high MAO-A enzymatic activity in the forebrain observed in this study can be dependent on MAO-A enzyme transported from the raphe to the forebrain region through the axons of the raphe population of serotonergic neurons.

In conclusion, this study found that acute social stress downregulates the expression of MAO-A at the transcriptional level and increases 5-HT metabolism. In addition, our data suggested the possibility of axonal transport of MAO-A enzyme. The acute social stress model using Nile tilapia would be useful to explore the mechanism underlying stress-induced changes in MAO-A transcription and 5-HT turnover rates.

## Author Contributions

IP, TS, and YH designed the research. YH performed the experiments and wrote the manuscript. IP and TS edited the manuscript.

## Conflict of Interest Statement

The authors declare that the research was conducted in the absence of any commercial or financial relationships that could be construed as a potential conflict of interest.
